# 3D-printed titanium implant with pre-mounted dental implants for mandible reconstruction: a case report

**DOI:** 10.1186/s40902-020-00272-5

**Published:** 2020-08-14

**Authors:** Jung-Hyun Park, Michidgerel Odkhuu, Sura Cho, Jingwen Li, Bo-Young Park, Jin-Woo Kim

**Affiliations:** 1 Department of Oral and Maxillofacial Surgery, Ewha Womans University Medical Center, Seoul, Korea; 2 Graduate School of Clinical Dentistry, Ewha Womans University, Seoul, Korea; 3 Department of Oral and Maxillofacial Surgery, School of Medicine, Ewha Womans University, Anyangcheon-ro 1071, Yangcheon-gu, Seoul, 07985 Korea; 4 Graduate School of Clinical Dentistry, Ewha Womans University, Seoul, Korea; 5 Department of Plastic Surgery, School of Medicine, Ewha Womans University, Seoul, Korea

**Keywords:** Titanium implant, 3D Printing, Mandible reconstruction, Osteoradionecrosis

## Abstract

**Background:**

This clinical case presented a novel method of segmental mandible reconstruction using 3D-printed titanium implant with pre-mounted dental implants that was planned to rehabilitate occlusion.

**Case presentation:**

A 53-year-old male who suffered osteoradionecrosis due to the radiation after squamous cell carcinoma resection. The 3D-printed titanium implant with pre-mounted dental implant fixtures was simulated and fabricated with selective laser melting method. The implant was successfully inserted, and the discontinuous mandible defect was rehabilitated without postoperative infection or foreign body reaction during follow-ups, until a year.

**Conclusions:**

The 3D-printed titanium implant would be the one of the suitable treatment modalities for mandible reconstruction considering all the aspect of mandibular functions.

## Background

Reconstruction of mandibular defect resulting from segmental mandibulectomy is one of many challenges faced by oral surgeons [1]. Segmental defect of the mandible, whatever the cause—benign tumor, malignant tumor, osteomyelitis, or osteoradionecrosis—impacts both facial esthetics and oral functions such as mastication, swallowing, and speaking. Many reconstruction modalities have been reported to achieve optimal functional and aesthetic results. Conventional modalities for mandibular reconstruction include reconstruction plate, microvascular fibula free flap, iliac bone graft, costochondral rib bone graft, and alloplastic prosthesis [2]. When the segmental defect is large, microvascular free flap has been the golden standard of mandibular reconstruction. It allows dental implant installation, enabling the recovery of mandibular function as well as mandibular shape and aesthetics. Autogenous graft, however, has its disadvantages such as donor site morbidity, extended operation time, and potential graft failure due to tissue necrosis [2].

Recent development in three-dimensional (3D) printing technology enabled fabrication of customized prosthesis. 3D-printed titanium implant has successfully been used for the reconstruction of facial bone defect including the mandible. The advantage of 3D-printed titanium implant is that it can be designed according to the defect size and morphology [3]. Customized titanium implants can be fitted accurately in the defective site without interference [4]. It allows for reduced operating time and also recovers the original contour of the mandible and facial symmetry. Even so, 3D-printed titanium implant has its limitations regarding oral function in occlusal rehabilitation.

The purpose of mandibular reconstruction is not only to re-establish the morphology of the lower third of the face but also to restore the patient’s oral function such as mastication and pronunciation [5]. This clinical case presents a novel method of mandible reconstruction using 3D-printed custom titanium implant in a patient who suffered from osteoradionecrosis due to radiation after squamous cell carcinoma resection. To rehabilitate dentition, we installed dental implants into the 3D-printed titanium mandibular implant. To the best of our knowledge, this is the first report on 3D-printed titanium implant with pre-mounted dental implants, attempting to overcome the limitations of dental implant-less titanium implant in dental rehabilitation.

## Case presentation

A 53-year-male was presented to Ewha Womans University Mokdong Hospital, diagnosed with squamous cell carcinoma of attached gingiva in the left mandible. After whole body examination including magnetic resonance images and positron emission tomography scan, metastasis to neck lymph node was confirmed, and the patient was staged cT4N1M0 by American Joint Committee on Cancer Cancer Staging. Wide excision and marginal mandibulectomy of the left mandible preserving the inferior border of the mandible as well as modified radical neck dissection were performed. For reinforcement, reconstruction plate was applied. Intraoral defect was closed with an anterolateral thigh flap. The permanent biopsy revealed tumor invasion to the inferior alveolar nerve; thus, postoperative radiation therapy with total dose of 60 Gy was performed 2 months after operation. After radiation therapy, the patient showed extra oral skin fistula on the left mandible. The fistula was closed using thoracodorsal artery perforator flap.

**Fig. 1:**
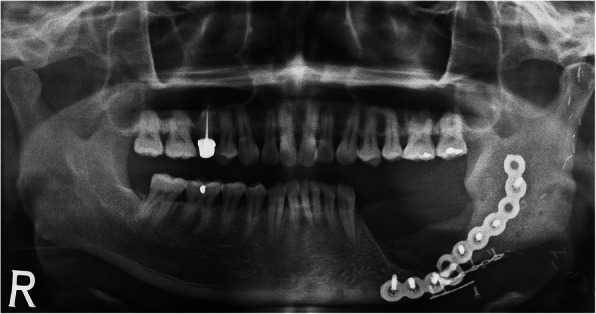
Pathologic fracture of the left mandibular body was observed in the panoramic radiograph

At the 1-year follow-up, the patient presented discomfort from changes in occlusion. Destructive bony change of the left mandibular body and pathologic fracture was observed in the panoramic radiograph and computed tomography (Fig. 1). Along with increased uptake on bone scintigraphy, the patient was diagnosed with osteoradionecrosis (ORN), and segmental mandibulectomy with reconstruction was planned for treatment. The patient was informed of the treatment options including autogenous microvascular free flap and 3D-printed titanium mandibular implant. The patient preferred a single surgical site to avoid donor site morbidity. Thus, customized 3D titanium implant, rather than the conventional fibula reconstruction, was selected for mandible reconstruction.

**Fig. 2:**
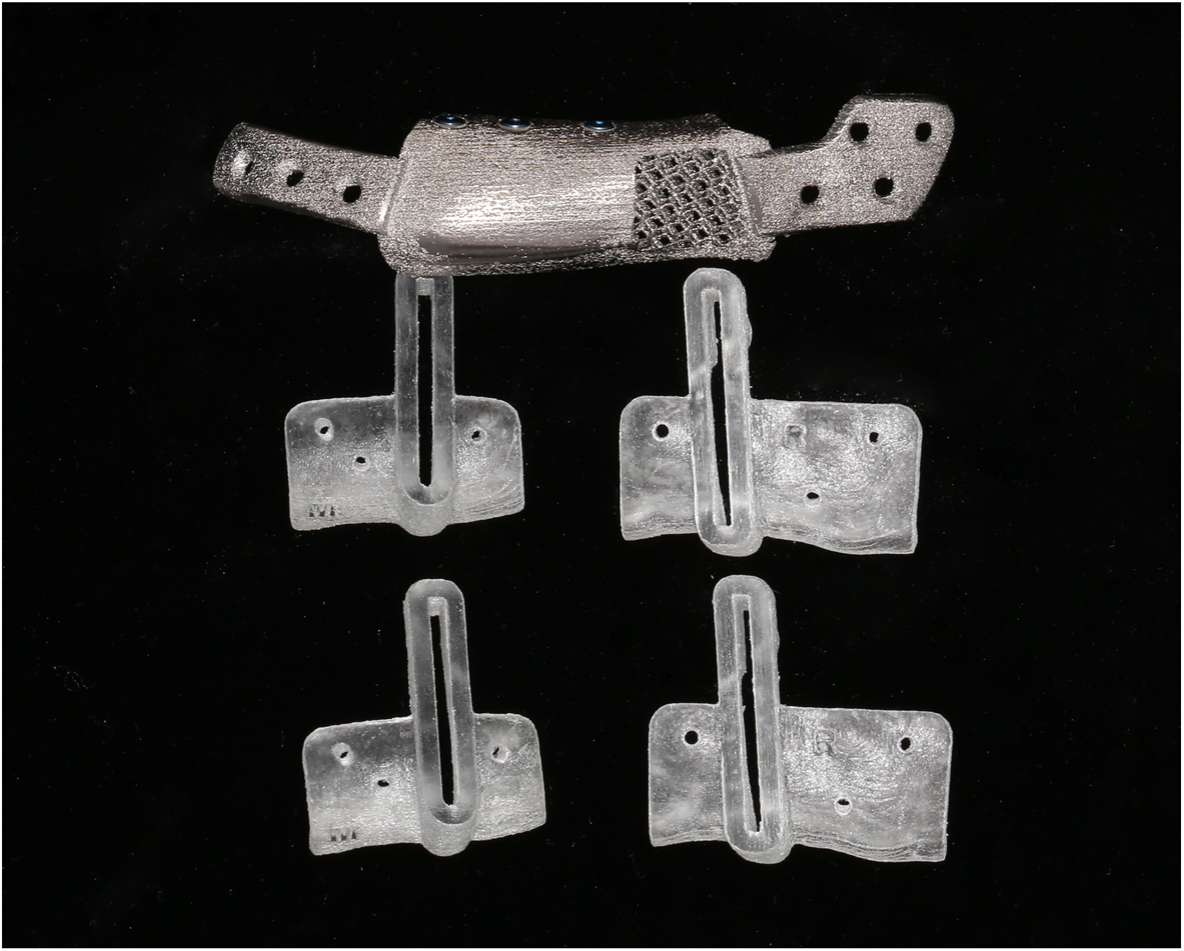
3D-printed resection guide and titanium mandibular implant

**Fig. 3:**
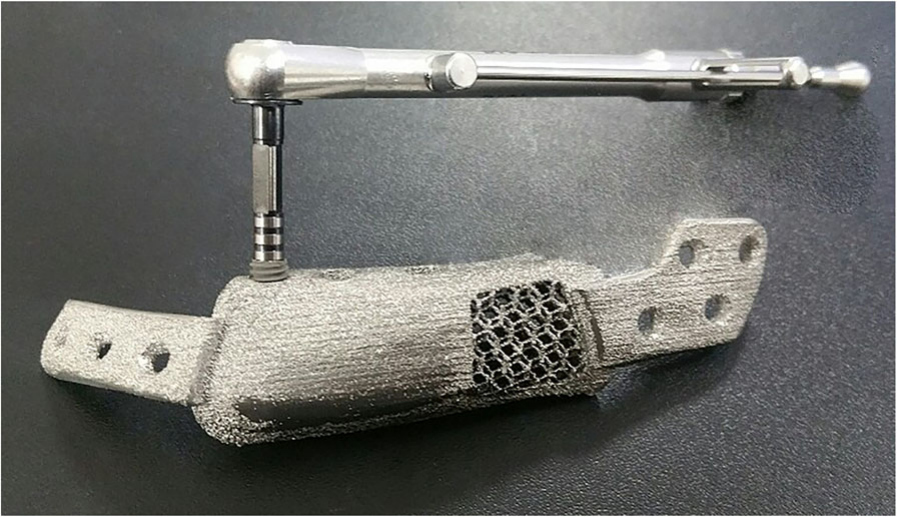
Dental implant fixtures were installed in the titanium mandibular implant

Pre-surgical simulation based on CT data was performed using image processing software (Mimics, Materialise, Belgium). The resection margin was carefully planned in accordance to the ORN lesion. Since the left proximal segment was rotated anterosuperiorly due to fracture, the segment was repositioned on the simulation using contralateral condylar position as the reference. The mandibular implant was designed to restore the original mandibular shape. Resection guide and titanium mandibular implant were fabricated using a 3D printer with selective laser melting (SLM) method (Fig. 2). All surface except bone contact surface of the titanium mandibular implant was polished. Bone contact surface was maintained rough to enhance osseointegration. Seven bicortical screw holes were designed on each ends of the implant for fixation. A portion of titanium implant distant from the dental implant insertion site was hollowed to decrease weight. For future dental rehabilitation, 3 dental implant fixtures (Dio implant, Busan, Republic of Korea) were installed in the titanium mandibular implant using conventional dental implant drilling system before the operation (Fig. 3). A titanium implant with only the female part of the fixture screw was first designed, but current SLM printing technology was unable to precisely reproduce the spiral thread of the small screw. Thus, we solely printed the titanium implant and manually installed the conventional dental implant to the titanium implant before surgery.

**Fig. 4:**
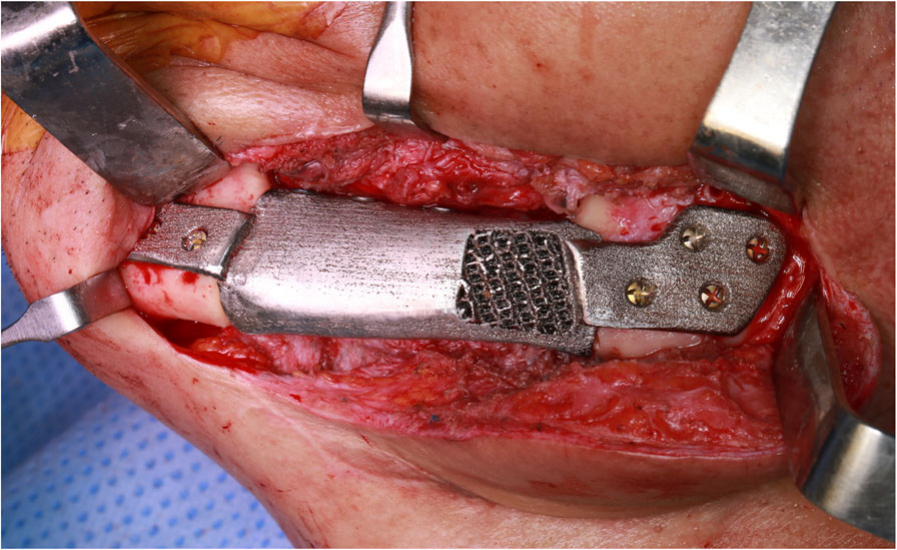
Titanium implant was inserted to the resected area of left mandible

Under general anesthesia, skin incision on the left neck was made through the existing scar. Pre-existing reconstruction plate and screws were removed, and the affected mandible was resected using 3D printed resection guides. Resection guides were well fitted on the mandible body according to the virtual plan. Subsequently, the titanium implant was inserted to the resected area and fixed using bicortical screws on the remaining mandible (Fig. 4). The dental implant was completely submerged inside the oral mucosa, and its exposure was planned in the subsequent second surgery. Recovery was uneventful, and the patient was discharged on the seventh day after surgery. At the 1-year follow-up after the operation, there were no evidences of postoperative infection or foreign body reaction. Pre-mounted implants were exposed during the second surgery for abutment placement, but the peri-implant mucosal healing was unfavorable; thus, the wound was closed, and implant exposure was re-planned for the future. The surgical protocol is available at https://www.youtube.com/watch?v=rd7FkbESRpA.

## Discussion

This clinical case presents a novel method of mandible reconstruction using 3D-printed titanium implant with pre-mounted dental implants to improve mandibular function. Recent application of 3D printing technology to medicine allows precise patient-specific preoperative design of implants. Surgeons are able to design the implant in ways to restore the original features, and greater implant stability is expected with an accurate fit in the defective site without interference [6]. 3D printing has many advantages over traditional methods such as its ability to fabricate complex structures, its improved customization, and its time efficiency.

3D-printed titanium implant has been used in various fields of facial reconstruction including the mandible for its mechanical strength that can support mandibular movement [6–8]. Unlike other facial compartments, reconstruction of the maxilla or mandible requires careful consideration of dental rehabilitation. Mastication and pronunciation are important functions of the mandible that can be restored with dental rehabilitation. The most widely used method for dental rehabilitation is the installation of dental implants to the vital bone for osseointegration. However, the use of titanium implant for occlusal rehabilitation is limited in that dental implants cannot be installed to the implant body itself.

There are several ways to install fixed prosthesis on titanium implants, one of which is via the abutment designed as part of the titanium implant [9]. Lee et al. reported the use of titanium mandibular implant with 2 abutment projections to rehabilitate occlusion [9]. Another possible way to install fixed prosthesis is by installing the conventional dental implant to the titanium implant as illustrated in this report. Because the exposure of titanium mandibular implant immediately leads to infection of the implant and eventual failure, dental implant insertion was done before the reconstruction surgery to avoid immediate exposure to the oral cavity. The concept of submerged dental implant procedure was applied. This aimed to allow initial healing period for osseointegration to the titanium mandible and promote barrier formation of soft tissue surrounding the titanium mandible including the periosteum. After this period, the dental implants were exposed to the oral cavity, and procedures for permanent prosthesis installation were carried out by the conventional method.

One of main goals of mandibular reconstruction is to restore the patient’s oral function such as mastication, swallowing, and pronunciation. The use of vascularized bone flap technique has been the golden standard for mandibular reconstruction treatment because it allows dental implant installation, promoting occlusal rehabilitation. Previously reported limitation of titanium implant was its inability for implant insertion [9], but in this report, 3D-printed titanium mandible implant was efficiently used in mandibular reconstruction with pre-mounted dental implant for occlusal rehabilitation. To avoid the risk of infection, the concept of submerged dental implant was applied. Although the soft tissues surrounding the titanium implant needs to be closely monitored after exposing the dental implant in the second surgery for any risk of infection, this case presents the possibility of conventional dental implant installation into titanium implant, which will play an important role in the rehabilitation of masticatory function for mandibular reconstruction.

## Conclusions

This case demonstrated the possibility of conventional dental implant installation into titanium implant for occlusal rehabilitation using the concept of submerged dental implant. 3D-printed titanium implant can be a suitable treatment modality for mandible reconstruction considering all aspects of mandibular functions.

## Data Availability

Data sharing is not applicable to this article as no data sets were generated or analyzed during the current study.

## Abbreviations

3D: Three-dimensional
ORN: Osteoradionecrosis
SLM: Selective laser melting
